# Defatted Black Soldier Fly Meal as a Dietary Protein Source for Grey Mullet (*Mugil cephalus*): Effects on Growth Performance, Gut Morphology, Spleen and Liver Health

**DOI:** 10.3390/ani16071012

**Published:** 2026-03-25

**Authors:** Basilio Randazzo, Letteria Caccamo, Stefano Carboni, Danilo Concu, Francesco Gai, Barbara Loi, Martina Meola, Andrea Miccoli, Simone Mirto, Alessandro Rinaldi, Anna Perdichizzi, Dario Vallainc, Giulia Maricchiolo

**Affiliations:** 1 Institute for Marine Biological Resources and Biotechnology, National Research Council, Via Spianata San Ranieri, 86, 98122 Messina, Italy; basilio.randazzo@cnr.it (B.R.); martina.meola@irbim.cnr.it (M.M.); anna.perdichizzi@cnr.it (A.P.); giulia.maricchiolo@cnr.it (G.M.); 2 National Biodiversity Future Center (NBFC), Piazza Marina 61, 90133 Palermo, Italy; s.carboni@fondazioneimc.it (S.C.); d.concu@fondazioneimc.it (D.C.); francesco.gai@cnr.it (F.G.); b.loi@fondazioneimc.it (B.L.); simone.mirto@cnr.it (S.M.); alessandro.rinaldi@cnr.it (A.R.); d.vallainc@fondazioneimc.it (D.V.); 3 International Marine Centre (IMC), Località. Sa Mardini, Torre Grande, 09170 Oristano, Italy; 4 Institute of Sciences of Food Production, National Research Council, L.go Paolo Braccini 2, 10095 Grugliasco, Italy; 5 Institute for Marine Biological Resources and Biotechnology, National Research Council, Largo Fiera Della Pesca 24, 60125 Ancona, Italy; andrea.miccoli@cnr.it; 6 Institute of Anthropic Impacts and Sustainability in Marine Environment, National Research Council, Lungomare Cristoforo Colombo 4521, Loc. Addaura, 90149 Palermo, Italy

**Keywords:** insect meal, fish nutrition, aquafeed, fish health, *Mugil cephalus*, *Hermetia illucens*

## Abstract

Subadult flathead grey mullets (*Mugil cephalus*) were fed three experimental diets containing increasing percentages of partially defatted black soldier fly (*Hermetia illucens*, BSF) that proportionally replaced the protein sources in the reference diet. At the end of the feeding trial, fish growth and gut, liver and spleen histology were evaluated. BSF inclusion did not significantly affect growth performances in any dietary groups. However, Fulton’s condition factor was lower in fish who were fed diets with the highest replacement levels (15% and 20%; BSF15 and BSF20) compared to those fed the 10% replacement (BSF10) and the control diet (BSF0). Histological analyses revealed increased villi thickness and mucous cell proliferation in the intestine of fish from BSF10 and BSF15 groups. A significant worsening of intestinal condition was observed in fish from the BSF20 group. Liver histology was not affected, while a dose-dependent effect on spleen was observed in fish fed BSF inclusions. Despite the absence of enteritis signs, an increase in macrophages/like TNF-α+ cells in the intestine mucosa indicated immune stimulation in the BSF10 group. Results indicate, for the first time, a threshold for BSF meal inclusion in diets specifically formulated for flathead grey mullets. The BSF10 diet was optimal, as growth performance was not affected and intestine health improved at both morphological and innate immune system levels.

## Simple Summary

In this study, *Hermetia illucens* meal was used as an ingredient in the diet of flathead grey mullet (*Mugil cephalus*) for the first time. This fish species is receiving increasing global attention due to its favourable biological characteristics, including low trophic feeding habits and high adaptability to diverse environmental conditions. In addition, its relatively low dietary protein requirement makes it a promising candidate for aquaculture diversification in the Mediterranean region, where carnivorous species still dominate production. Our results identified an inclusion threshold for *H. illucens* meal in the *M. cephalus* diet. Above this threshold, gut and spleen histology were negatively affected, likely due to the high chitin content of the ingredient. In contrast, low inclusion levels did not impair growth and promoted stimulation of the intestinal innate immune response. We conclude that *H. illucens* meal represents a suitable dietary ingredient for flathead grey mullet when included at low levels.

## 1. Introduction

Over recent decades, aquaculture has expanded to meet the global demand for seafood, while widespread efforts have aimed to reduce its impact on natural resources [1,2]. The feed sector has often been recognized as having a major ecological footprint. Consequently, aquafeed formulations have increasingly incorporated terrestrial protein sources, such as soybean meal (SBM), to reduce reliance on overexploited marine ingredients, particularly fish meal (FM) [3,4,5]. However, despite reductions in FM inclusion levels, the overall increase in aquaculture production has prevented a substantial decrease in its total use [6,7]. In the Mediterranean area, carnivorous fish culture represents the predominant production activity, with the gilthead seabream (*Sparus aurata*) and the European seabass (*Dicentrarchus labrax*) being the two major commodities [8,9]. These species are highly dependent on marine-derived ingredients, thus limiting the extent of FM substitution in the diets, and, consequently, reducing the potential sustainability improvements of new feed formulations. Therefore, diversifying farmed fish species, particularly by promoting those with low dependence on FM, may represent a complementary strategy to further reduce the use of limited natural resources [10]. Improving aquaculture sustainability and resilience will thus involve cultivating lower-trophic-level fish species with a marked tolerance for a wide range of environmental conditions, making them more suitable for future climate scenarios [11,12]. The flathead grey mullet (*Mugil cephalus*) is an euryhaline, omnivorous fish, distributed worldwide in temperate and tropical coastal waters [13]. It has high commercial value, not only for its flesh, but also for its salted and dried roe, known as “bottarga”—a high-priced delicacy appreciated in different countries [14]. Traditionally, *M. cephalus* farming is conducted in extensive monoculture and polyculture systems where feeding relies on natural productivity and/or on the partial integration with extruded pellet designed for other fish species (i.e., tilapia and common carp) [15,16,17,18,19]. Only recently have efforts shifted toward formulating practical species-specific diets for this species, with a focus on sustainability [20]. *M. cephalus* is an omnivorous, opportunistic feeder, and its protein requirement is lower than that of strictly carnivorous species such as seabass and gilthead seabream [21,22]. However, knowledge on its nutrient requirements is scarce and limited to the lipid and protein fractions, estimated as 6% and 30%, respectively [23,24,25,26]. The few available commercial feeds specifically formulated for this species are based on wheat flour, SBM and FM as main protein sources [17,18,19,27,28,29,30]. Over the last decades, a set of novel protein sources have been explored as potential FM substitutes in diets for *M. cephalus*. However, while some sources, including seaweeds, algae and dried zooplankton, have been shown as suitable for replacing a large percentage of FM in the diet with positive effects on feed utilization, growth and gut health [28,31,32], others have negatively affected fish performance. For instance, dietary inclusion of single-cell protein from *Corynebacterium glutamicum* resulted in impaired fish growth performance, and inhibited alkaline protease and aminopeptidase enzymatic activity [20]. The exploration of novel protein sources for this species is in its infancy, and most studies to date have been carried out on larval or juvenile stages.

Insects have been thoroughly evaluated as a valuable protein source in fin fish diets, particularly considering the EC Regulation No. 893/2017 which authorized their use in fish nutrition [33,34]. More specifically, the black soldier fly (*Hermetia illucens*, BSF) has gained considerable interest due to its low ecological footprint, high protein content, and balanced essential aminoacidic profile that is rich in tyrosine, phenylalanine and histidine compared to other feedstuff [35,36,37,38]. Nonetheless, the extensive use of this promising ingredient is still hampered by its not yet competitive cost (around 10 USD per Kg). On the other hand, BSF meal can be used at low percentages as a nutraceutical ingredient in aquafeeds, as it contains several bioactive compounds such as chitin, antimicrobial compounds, and medium-chain fatty acids that exert positive effects on fish health [39,40,41].

Chitin in particular is known as a prebiotic that can induce microbiome-mediated beneficial activity at gut level in different fish species [42,43,44]. Similarly, lauric acid (C12), a medium-chain fatty acid (FA) that largely characterizes BSF composition, is known for its anti-inflammatory effects on the gut of many fish species [45,46,47]. Several studies have explored the use of BSF meal-based diets for different life stages of a number of fish species [48,49,50,51,52,53,54,55,56,57,58,59]. The present study was carried out to assess whether BSF meal can be used to partially replace conventional protein sources in diets specifically formulated for flathead grey mullet. To the best of our knowledge, no study using BSF in diets for this fish species has been performed to date. However, due to limited knowledge about flathead grey mullet tolerance towards novel ingredients, the use of BSF should be carefully considered, particularly given the peculiar feeding habits of this fish species and the BSF chitin content, which may impair growth and nutrient digestibility when included at high doses. For these reasons, in the present study, low-to-medium BSF dietary inclusions were tested to provide first insights into the physiological responses of flathead grey mullet to this ingredient. Three BSF inclusion levels were tested, and at the end of the experiment, the effects on fish growth and gut, spleen and liver health were assessed.

## 2. Materials and Methods

### 2.1. Ethics

The feeding trial experiment and all the procedures involving animals were carried out in strict accordance with EU legal frameworks relating to the protection of animals used for scientific purposes (Directive 2010/63/EU). It was approved by the Italian Ministry of Health (n. 5598B.N.T61).

### 2.2. Experimental Diets

Four practical pelleted diets were formulated to be approximately iso-proteic, iso-lipidic and isoenergetic, as reported in Table 1. A control diet (BSF0) containing only conventional protein sources was used as a reference, based on diets formulated by Bertini et al. [20]. Three experimental diets were formulated by replacing 10%, 15%, and 20% (of the conventional protein sources in the reference diet—fish meal (FM), poultry meal (PM), feather meal (FtM), and soybean meal (SBM)—with partially defatted BSF prepupae meal, resulting in experimental diets named BSF10, BSF15 and BSF20. Conventional protein sources, which accounted for 48% of the control diet (BSF0) on a dry matter basis, were proportionally replaced with increasing levels of BSF meal. In each experimental diet, the substitution was made by including BSF meal at expenses of FM, PM, FtM, and SBM in equal amounts across the four ingredients (grey background in Table 1). All the ground ingredients and oils were individually weighted and mixed with a blender (Brevetti S.A.G.A, Milano, Italy). The pelleting process was performed using a meat grinder and 1.5 mm pellets were subsequently dried (50 °C for 48 h) and stored at −20 °C until used. Feed samples were analyzed in duplicate for dry matter (AOAC#934.01), crude protein, total nitrogen (N x 6.25) (AOAC #984.13), and ash (AOAC #942.05) contents according to AOAC International [60]; ether extract content was analyzed according to AOAC International [61]. Gross energy content was measured by an adiabatic bomb calorimeter (IKA C7000, Werke GmbH and Co., Staufen, Germany). The approximate composition of the diets and their essential aminoacidic composition are shown in Table 1 and Table S1, respectively.

### 2.3. Fish Rearing, Feeding Trial and Sampling

Three hundred and eighty juvenile grey mullets (weight = 40.2 ± 0.5 g) were obtained from the International Marine Centre (IMC, Oristano, Italy) and transferred to the aquaculture experimental facility of the Institute for Marine Biological Resources and Biotechnology of the Italian National Research Council (IRBIM-CNR, Messina, Italy). After one week of acclimation, 20 randomly chosen specimens were sacrificed with an overdose of anesthetic (MS222, Tricaine Pharmaq (Oslo, Norway), 500 mg L^−1^) to assess the initial health conditions by visual inspection and histological analyses of target organs, as described in Section 2.5. The remaining fish (n = 360) were randomly divided in 12 experimental tanks (n = 30 each tank). Tanks (volume 1.4 m^3^) were connected to a flow-through system in which incoming seawater was continuously pumped, sand-filtered and sterilized using an UV lamp; flow rate was set to guarantee 100% tank volume exchange per hour, and a natural photoperiod was adopted. Fish were assigned to the four dietary treatments in triplicate and fed for 138 days. Feeding ratio was adjusted in all the treatments according to the water temperature (1–3% body weight). Fish from all tanks were bulk-weighted monthly to adjust the amount of feed per tank. Fish were hand-fed in three daily meals (8:00 a.m., 12:00 and 4:00 p.m.). Water parameters were measured daily (temperature, 20.5 ± 9.12 °C; O2, 66.5 ± 8.1 mg L^−1^; salinity, 39.07 ± 0.41 PSU; pH, 8.62 ± 0.42). Experimental tanks were cleaned daily by syphoning to remove feces and uneaten feed. At the end of the feeding trial, after euthanasia (MS-222 500 mg L^−1^), fish were individually weighted and target organs were sampled as described below.

### 2.4. Growth Performances and Somatic Indexes

At the end of the experiment, fish were individually measured and weighted to calculate the following growth and somatic indexes:

Specific Growth Rate SGR;%d−1=ln(final body weight−lninitial body weightdays  of feeding∗100

$$Feed\;conversion\;ratio\left(FCR\right)=\frac{feed\;supplied}{weight\;gain}*100$$

$$Hepatosomatic\;index\left(HSI;\%\right)=\frac{liver\;weight}{body\;weight}*100$$

$$Viscerosomatic\;index\left(VSI;\%\right)=\frac{viscera\;weight}{body\;weight}*100$$

$$Fulton’s\;condition\;factor\left(K\right)=\frac{body\;weight}{total\;length^{3}}*100$$

### 2.5. Histology and Morphometric Evaluations

Twelve specimens per dietary group (n = four per tank) were used for histological analyses. Briefly, after dissection, intestine (portions of anterior and posterior tracts), spleen and liver samples were immediately fixed in Bouin’s solution for 24 h. Samples were then dehydrated through graded ethanol solutions, clarified in xylene (Bio-Optica, Milan, Italy) and embedded in paraffin blocks. Histological sections (5 µm thickness) were obtained using a rotatory microtome, placed on glass slides for histological staining (H and E), and observed using a Leica DFC 295 light microscope (Leica, Wetzlar, Germany). Images were acquired using a combined colour camera (Optika Microscopes, Bergamo, Italy, C-HB model). For morphometric evaluations of the intestinal villi length and thickness, three sections per sample were considered, and measurements were performed using the Leica images analyzer (Leica IM1000 Image Manager v.1.20); all the undamaged and entire villi in each section were measured. For goblet cell quantification, histological sections were stained with Alcian Blue (pH 2.5, Bio-Optica, Milan, Italy) and three sections per sample were photographed at 20× magnification. All the goblet cells in the randomly chosen photographic fields (one per section), corresponding to 304.000 μm^2^, were counted. Goblet cell distribution was homogeneously spread throughout the intestinal mucosa, and photographic fields were chosen to avoid areas with artefacts. Histopathological analysis of intestinal condition was performed using double-blind evaluation to attribute a semi-quantitative score, according to the scoring system adapted by Uran et al. [62], Penn et al. [63] and Garcìa-Ortega et al. [64], and considering the following parameters: edema, epithelium detachment, enterocytes nuclei delocalization, enterocytes supranuclear vacuolization, submucosa thickening, thickening of lamina propria, and inflammatory infiltrate. For each parameter, a four-point score was assigned, as reported in Supplementary Materials (Table S2). Results were reported as the mean of the sum of the score for each parameter. For the spleen, three randomly chosen microscopic fields (38.800 µm^2^) per section were acquired for the total count of melanomacrophage centres (MMCs). MMC count was performed on the total of the MMCs observed. In addition, MMCs were counted based on the area of the aggregates as follows: small (up to 20 µm^2^), medium (20–80 µm^2^), and large (over 80 µm^2^). Moreover, the incidence of splenic parenchyma alteration—including loosening of the splenic parenchyma, reduced discrimination between white and red pulp, expansion of white pulp with increased lymphocytes, and a reduction in red pulp—was evaluated as a percentage of the total of samples analyzed. For the examination of the liver samples, three microphotographs were acquired at a magnification of 40× on the central portions of each section; the hepatocytes and the decentralized nuclei present in the photographed areas (76,000 μm^2^ each) were counted in each specimen.

### 2.6. Immunohistochemistry

Twelve specimens per dietary group (n = four per tank) were used. Samples were processed and embedded in paraffin blocks as described in Section 2.5. Paraffin sections (7 µm thickness) were placed on poly-L-lysine coated slides (Bio-Optica, Milan, Italy) to prevent detachment; three sections per sample were used for the two antibodies considered (i.e., anti-IL-1β and anti-TNF-α). After deparaffinization and re-hydration, sections were treated with 0.3% hydrogen peroxide for 5 min at room temperature to inhibit endogenous peroxidase activity. Then, antigen retrieval was performed in tris-EDTA buffer (10 mM Tris base, 1 mM EDTA solution, pH 9) using microwave oven at 800 W for 3 min. Sections were then cooled for 15 min at room temperature (RT) in 0.01 M phosphate-buffered saline (PBS), pH 7.4. To prevent non-specific antibody binding, slides were incubated in 20% BSA for 20 min. Sections were then incubated overnight at 4 °C with mouse monoclonal anti-IL-1β (E7-2-hIL1β:sc-32294, Santa Cruz Biotechnology, Inc. Dallas, TX, USA) and rabbit polyclonal anti-TNF-α (ab6671, Abcam, Cambridge, UK), both at 1:100 dilution. Slides were then rinsed with PBS and incubated at RT for 1.5 h with the secondary peroxidase-conjugated antibodies, namely Goat Anti-Mouse (A9917, Sigma-Aldrich, Saint Louis, MO, USA) for anti-IL-1β, and 1:200 Goat Anti-Rabbit (ab6671, Abcam) for anti-TNF-α, both at 1:200 dilution. The peroxidase reaction was developed in a solution of 3, 3-diaminobenzidine tetrahydrochloride (Sigma; 0.04% *w*/*v* in Tris−HCl 0.05 M, pH 7.4) and H_2_O_2_ (0.005%). Staining was indicated by brown coloration. Negative controls were obtained by incubation without the primary antibody.

All IL-1β+ and TNF-α+ cells in each section were considered for intestinal quantification. For splenic samples, cells were counted in a central 2 mm^2^ area on each section to assess an equal area across all sections. Results were reported as mean ± standard deviations of the counts. To better identify cell types, immediately adjacent sections were stained with H&E, as described in Section 2.5.

### 2.7. Statistical Analyses

Final body weight (BW), growth performance parameters (SGR and FCR), and morphometric indices (HSI, VSI, and K-Fulton) were analyzed using one-way analysis of variance (ANOVA), with diet as fixed factor (four levels). Statistical analyses were performed considering the tank as the experimental unit (n = three tanks per dietary treatment). Individual fish measurements were averaged within each tank prior to statistical analysis to avoid pseudo-replication.

Assumptions of normality and homogeneity of variances were assessed using the Shapiro–Wilk test and Levene’s test, respectively. When significant differences (*p* < 0.05) were detected in the ANOVA model, Tukey’s test (HSD) was applied for pairwise comparisons.

Survival data (expressed as percentage) were arcsine square-root transformed prior to analysis; however, since the assumption of normality was not satisfied, differences among dietary treatments were evaluated using the non-parametric Kruskal–Wallis test.

Results are presented as mean ± standard error (SD). All analyses were performed using R software (version 4.5.0; R Core Team, 2024) with the following packages: car, DescTools, agricolae, ggplot2, dplyr, and readxl.

For the statistical analyses of the histological results on intestine, spleen and liver, and for the count of the immunohistochemically marked cells, the Graph software package Prism5 (Graph Pad Software, version 9.3.1, La Jolla, CA, USA) was used. Results were reported as mean and standard deviation (SD) of the observations and were analyzed through one-way ANOVA with pairwise post hoc comparison to assess the impact of the diet on the different parameters considered. The homogeneity of variance was examined using Levene’s test. In case of homoscedasticity, the standard parametric ANOVA was applied with Tukey’s post hoc comparisons, while in the case of heteroscedasticity, a nonparametric ANOVA with Games–Howell post hoc comparisons was adopted. In all tests, a *p*-value equal to or less than 0.05 was considered statistically significant.

## 3. Results

### 3.1. Growth Performances, Somatic Indexes and Survival

Fish promptly accepted the experimental diets, and a mortality rate of less than 0.5% was recorded in all tanks without statistically significant differences among groups (Kruskal–Wallis test, H = 3.483, *p* = 0.323). At the end of the feeding trial, all experimental groups exhibited a doubling of the initial body weight (BW) (Table 2). Fish from all treatments showed a clear increase in BW. Although the final BW did not differ significantly among groups (ANOVA, *p* = 0.064), a trend toward treatment-related variation was observed. No statistically significant differences in FCR and SGR were observed among the experimental groups. Conversely, Fulton’s condition factor (K) was significantly affected by the dietary treatments (*p* = 0.019). Post hoc analysis showed that fish in the BSF20 group had significantly lower K values than those in the BSF10 group (*p* = 0.032), whereas the BSF0 group exhibited significantly higher K values than the BSF20 group (*p* = 0.024). No statistically significant differences in HSI and VSI were observed among the groups.

### 3.2. Histology

#### 3.2.1. Intestine

**Figure 1:**
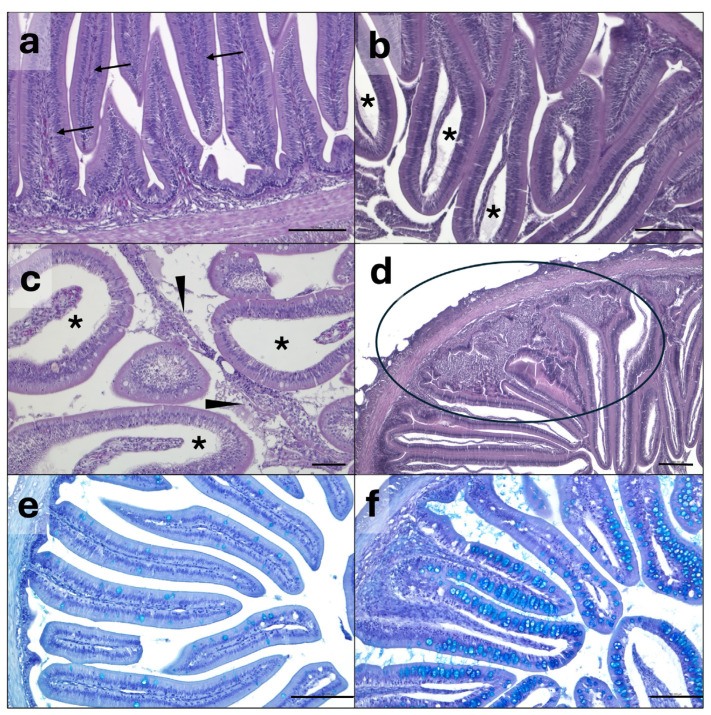
Representative histological pictures of anterior intestine showing some of the morphological alterations used for intestine condition evaluation by mean of the enteritis semi-quantitative score system. Correctly aligned nuclei (arrow) and no enteritis signs in BSF0 group (**a**). Edema (asterisks) and epithelial detachment (arrowhead) in anterior intestine from BSF15 (**b**) and BSF20 (**c**) groups. Inflammatory infiltrate (circle) in group BSF20 (**d**). Goblet cell (light blue staining) distribution in BSF10 (**e**) and BSF20 (**f**) groups. Hematoxylin and eosin (**a**–**d**). Alcian blue (**e**,**f**). Scale bar: (**a**,**b**,**d**–**f**) = 100 µm; c = 50 µm.

Visual and histological examination at the beginning of the experiment did not reveal pathological conditions in any of the samples analyzed. Results of the morphometric measurements and condition evaluations by enteritis score in the intestine are summarized in Table 3 and Table 4, respectively. Statistically significant differences between groups were evident in the morphometric and enteritis score analyses of the anterior intestine, while no differences (*p* > 0.05) were observed in the posterior intestine. Specifically, villi thickness was significantly higher in the anterior intestine of fish from the BSF10 and BSF15 groups (*p* = 0.011), while mucous cells were more abundant (*p* = 0.001) in the BSF15 group compared to the BSF0 group. Intestine condition evaluation revealed a significantly higher enteritis score in the anterior intestine of fish from the BSF20 group compared to the other groups (*p* = 0.02). Representative microphotograph fields of intestine from the different experimental groups, at the end of the experiment, are reported in Figure 1.

#### 3.2.2. Liver

**Figure 2:**
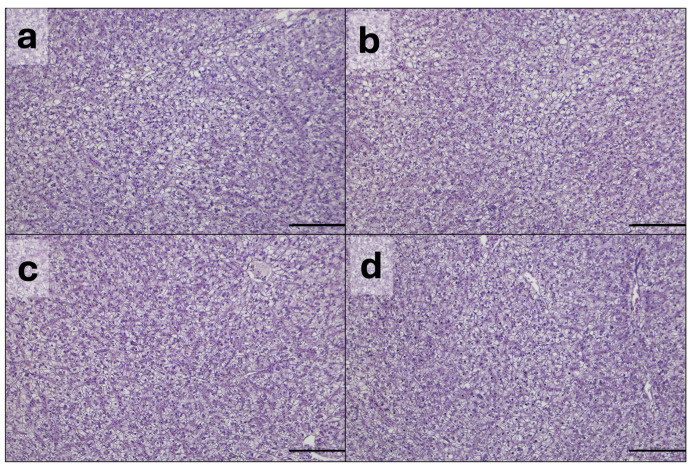
Representative histological pictures of liver from fish fed BSF0 (**a**), BSF10 (**b**), BSF15 (**c**) and BSF20 (**d**) diets. Hematoxylin and eosin. Scale bar = 100 µm.

Results of the hepatocyte and decentralized nuclei counts per area are summarized in Table 5. No statistical differences among groups were found in any of the parameters evaluated (*p* > 0.05). Representative histological pictures of liver from the different experimental groups are reported in Figure 2.

#### 3.2.3. Spleen

**Figure 3:**
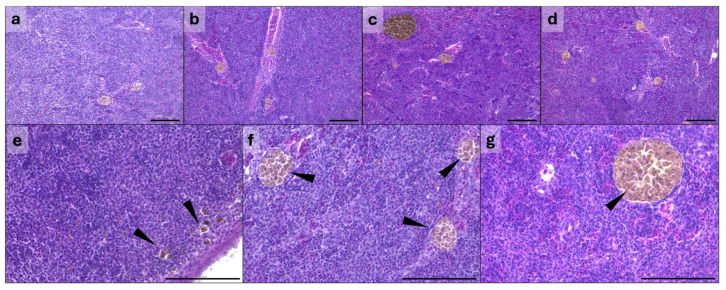
Representative histological pictures of spleen of fish fed BSF0 (**a**), BSF10 (**b**), BSF15 (**c**) and BSF20 (**d**) diets. Melanomacrophage centre (arrowhead) size classes: small (**e**), medium (**f**) and large (**g**). Hematoxylin and eosin. Scale bar = 100 µm.

Representative histological pictures of spleen from all the experimental groups are shown in Figure 3a–d. All spleen samples analyzed from BSF0 group showed normal histological architecture with a normal ratio of white pulp, consisting of aggregated lymphoid cells, to red pulp, containing mainly red blood corpuscles. In contrast, histological changes in splenic tissue were observed in the BSF10, BSF15 and BSF20 groups. These changes included loosening of the splenic parenchyma (an indistinct boundary between white pulp and red pulp), expansion of the white pulp with increased lymphocytes, and shrinkage of the red pulp with reduced red blood cells. The incidence of these changes was observed in 0%, 38%, 57% and 80% of the samples analyzed in the BSF0, BSF10, BSF15 and BSF20 groups, respectively.

Results of the melanomacrophage centre (MMC) count are summarized in Table 6. The total number of MMCs did not show significant differences among treatments (*p* > 0.05). In contrast, statistically significant differences were observed when considering MMC size class. Particularly, a significantly higher number of small MMCs were observed in the BSF20 group compared to the other experimental groups (*p* = 0.001). Large MMCs were predominant in the BSF10 and BSF15 groups compared to BSF0 and BSF20 (*p* = 0.001). In Figure 3e–g, a representation of the different MMC size classes considered for the analyses is presented.

### 3.3. Immunohistochemistry

**Figure 4:**
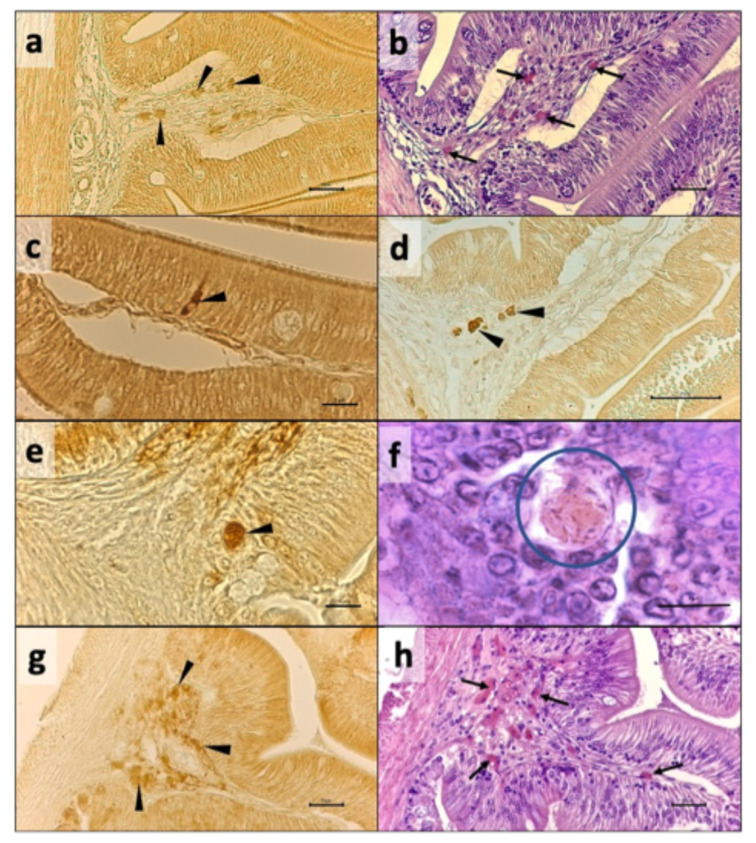
Representative microphotographs of cells marked by immunohistochemistry with anti-IL-1β and anti-TNF-α antibodies, and adjacent sections stained with hematoxylin and eosin (H and E). (**a**) IL-1β+ cells in the lamina propria of the anterior intestine from the BSF0 group (arrowhead) and (**b**) adjacent section showing eosinophilic stain (arrow) in cells with the same distribution pattern of IL-1β+ ones; (**c**) intraepithelial, finger-shaped enteroendocrine IL-1β+ cell (arrowhead) intercalated within the absorptive mucosal layer; (**d**) strongly marked IL-1β+ cells (arrowhead) in the posterior intestine from the BSF15 group. (**e**) Round-shaped TNF-α+ cells (arrowhead) at the base of a mucosal fold in anterior intestine from BSF10 group and (**f**) H and E-stained adjacent section showing higher magnification of a slightly eosinophilic round-shaped cell (circle). (**g**) TNF-α+ cells (arrowhead) in lamina propria in the anterior intestine from the BSF20 group, and (**h**) H and E-stained adjacent section showing eosinophilic cells with a similar distribution pattern of TNF-α+ ones (arrow). Scale bar: (**a**,**b**,**d**,**g**,**h**) = 50 µm; **c**, **e**, **f** = 5 µm.

Representative microphotographs of IL-1β+ and TNF-α+ cells in intestine are reported in Figure 4. No immunopositivity was observed in the negative sections. IL-1β+ and TNF-α+ cells were found in the intestinal lamina propria of all samples analyzed. These cells were mostly eosinophilic and globular, as revealed by the H and E staining of adjacent sections (Figure 4b,f). Moreover, intraepithelial, finger-shaped enteroendocrine cells were also marked by anti-IL-1β antibody (Figure 4c). Neutrophil-like, round-shaped TNF-α+ cells were also observed (Figure 4h). IL-1β+ cell counts in either the anterior or posterior tracts were not significantly different among groups (Table 7). In contrast, a significantly higher number of TNF-α+ cells (*p* = 0.0015) was observed in the anterior intestine of fish from the BSF10 group. In the posterior intestine, no clearly marked TNF-α+ cells were observed in any of the sections analyzed.

## 4. Discussions

The present study evaluated, for the first time, the effects of graded inclusion levels of black soldier fly meal as a dietary protein source in flathead grey mullet (*Mugil cephalus*), assessing growth performances and physiological responses in comparison with a reference diet specifically intended for this fish species. The results here obtained showed that a 10% replacement of conventional protein ingredients with BSF prepupae meal (BSF10) did not negatively affect fish zootechnical parameters (i.e., it had no adverse effect on growth performance). Moreover, no statistically significant differences in final body weight, FCR or SGR were observed among treatments up to the 20% inclusion, although a tendency toward reduced final body weight was detected at higher inclusion rates. Only Fulton’s condition factor (K) was significantly affected by dietary treatment, with lower values recorded in the BSF20 group. This suggests a possible alteration in nutrient allocation rather than a clear impairment of growth performance. Although not considered an important economic trait, Fulton’s condition factor is an important tool in dietary trials. It is used to assess fish nutritional status, nutrient allocation efficiency in muscles, and overall fish condition [65,66]. To date, several studies have evaluated the use of BSF meal as a protein source in diets for several fish species, with a heterogeneous range of physiological species-specific responses [57]. In this regard, partial to almost total replacement has been shown to be tolerable for many fish species, while deleterious effects on growth and feed efficiency were observed in others (for a review, see Hua et al. [67]).

One common explanation for the negative effects on physiology of fish fed high BSF inclusions involves the chitin content of insect meals, ranging between 6 and 9% on dry matter in prepupae stage [68,69,70]. Chitin is considered responsible for reducing feed digestibility in some fish species [55,71,72,73,74,75,76,77,78], as the ability of fish to digest chitin varies greatly depending on chitinolytic enzyme activity [72]. In this regard, investigating chitinolytic enzyme activity in flathead grey mullet would be useful to clarify the actual role of chitin in inducing the physiological effects observed in our study. Although no studies have specifically addressed chitinolytic activity in *M. cephalus*, its adult feeding strategy, primarily based on detritus and organic debris, may indicate a lower enzymatic capacity to break down this polysaccharide [79,80]. Beside these explanations, additional factors may contribute to the observed outcomes. While the nutritional requirements (amino acids, fatty acids, vitamins and micronutrients) for many farmed species have been mostly established, knowledge on *M. cephalus* remains limited [23,24,25,26,81,82]. However, the reliance of *M. cephalus* on fish meal (FM) is significantly lower compared to other species, thus not leading to assumed deficiencies related to low dietary FM in our study. Previous studies showed that the replacements of up to 75% FM with plant-derived proteins and up to 20% of *Ulva* sp. or yeast do not compromise the growth and digestive physiology in *M. cephalus* fingerlings [29,31]. Other studies demonstrated that 100% FM replacement with dried zooplankton biomass resulted in improved growth performances, feed utilization and gut health in *M. cephalus* larvae and juveniles [28,32]. In contrast, substitution of conventional dietary protein sources with 25% *Saccharomyces delbrueckii* or bacterial single-cell proteins was linked to reduced growth performances of mullet fingerlings [20,83]. In our study, no severe effect on fish growth was related to BSF dietary inclusion; thus, the effects on Fulton’s condition factor should be considered cautiously.

As for the intestine, only marginal effects of the test diets were detected. Thus, severe intestinal tissue damage responsible for the impairment in fish growth can be ruled out, at least when fish were fed up to 15% replacement of the total amount of four conventional ingredients (FM, PM, FtM and SBM) with BSF meal. Changes were primarily observed in the anterior intestinal tract, which in this study appeared more responsive than the posterior one. Indeed, the increase in villi thickness in the higher replacement treatments indicates an enlargement of the mucosal absorptive surface but in the absence of inflammation onset, as supported by the enteritis score results. On the other hand, the increase in mucous cell abundance observed in the anterior intestine of the BSF15 group could be ascribed to a mucous-inducing effect of BSF meal, similar to what has been observed in other fish species including rainbow trout (*Onchorhinchus mykiss*), barramundi (*Lates calcarifer*), Nile tilapia (*Oreochromis niloticus*), clownfish (*Amphiprion aocellaris*) and Atlantic salmon (*Salmo salar*) [84,85,86,87,88].

Remarkably, a significant decline in intestinal condition was observed in the BSF20 group, as revealed by the enteritis semi-quantitative analyses, the lack of mucous cell proliferation, and the worsening of growth performance (K factor, in particular). This suggests a dietary threshold for BSF meal inclusion for this species, beyond which gut health and somatic conditions may begin to be compromised, even in the absence of overt growth depression. Furthermore, in the present study, the spleen was considered by virtue of its importance as an immuno-competent lymphoid organ [89]. Histopathological analyses of spleen revealed appreciable differences in tissue structure in relation to BSF meal inclusion. These changes mainly consisted in the loosening of the splenic parenchyma, reduced distinction between white and red pulp, expansion of white pulp with increased lymphocytes, and a reduction in red pulp, and were more pronounced in the BSF20 group, suggesting a systemic dose-dependent effect. Beside chitin, BSF meal contains a set of bioactive compounds, including medium-chain fatty acids, which have been shown to exert modulatory effects on fish immune responses [39,41,56]. Moreover, splenic melanomacrophage centres (MMCs) were analyzed, as they have both physiologic and pathological functions in fish and are involved in immune responses [90]. The reduction in MMC size observed in the BSF20 group deserves further investigation to elucidate whether any component of BSF meal, including chitin, negatively affects immune functions beyond a certain threshold of inclusion, as previously observed in other fish species [33,56,75,76]. In contrast, the enlarged MMCs observed in the BSF10 and BSF15 groups support the role of BSF meal bioactive compounds in stimulating innate immunity, promoting macrophage activation, and pro-inflammatory cytokine production, as previously observed [44], and are consistent with enhanced innate immune stimulation [91]. Recent studies on *Poecilia sphenops* highlighted the role of MMCs in immune responses, including phagocytosis and detoxification [92]. The MMC size-class reduction observed in the BSF20 group might indicate a less effective immune response or a potential negative effect of high chitin levels, leading to immunity impairment.

Finally, immunohistochemical analyses of the intestine were performed to detect the abundance of cells expressing IL-1β and TNF-α, which are key cytokines involved in immune mechanisms, including in response to nutritional factors [93,94]. Generally, both antibodies used presented similar affinity to macrophage-like, round-shaped cells in the intestinal lamina propria, while anti-TNF-α also strongly marked intraepithelial enteroendocrine cells, which were also considered. Similar to what was observed in the histological analyses, differences were only observed in the anterior intestine, where a significantly higher abundance of macrophage-like TNF-α+ cells was found in the BSF10 group. Since no severe enteritis signs or other tissue morphological alterations were detected in this experimental group, the higher presence of TNF-α+ cells should not be attributed to inflammation onset, but rather to an enhancement in mucosal innate immune defence. Macrophages are highly plastic functional cells involved in host defences and inflammatory/regulatory mechanisms that have garnered much attention in various fish models [95]. These leukocytes undergo activation under a cascade of molecular mediators, including TNF-α, which is also considered a marker for macrophage maturation [96]. In this regard, in koi carp (*Cyprinus carpio* var. koi) an immunomodulatory effect was observed in relation to dietary BSF meal inclusion, resulting in an increased mRNA level for key regulator cytokines, including TGF-β, IL1, IL10, and TNF-α [97]. The immunomodulatory potential of BSF meal in fish diet has been observed in different fish species, with a dose-dependent and species-specific effect [98,99]. Particularly, the effects observed on the biological mechanisms involved in immune responses have been mainly attributed to the presence of bioactivity compounds, including chitin, which, when used at low concentrations, has been reported to enhance cellular innate immunity through microbiota-mediated mechanisms [72,100]. Low dietary inclusions of chitin have been shown to exert beneficial effects on health in some fish species, acting as a prebiotic and immunostimulant ingredient [33,56,93,101,102,103]. Moreover, BSF meal is particularly rich in lauric acid, a medium-chain fatty acid demonstrated to exert immuno-boosting macrophage-mediated properties in the gut of vertebrates, including fish [104,105].

## 5. Conclusions

The results obtained in the present study demonstrated that up to 10% substitution of dietary conventional protein sources with BSF meal can be successfully applied in diets for flathead grey mullet. Although growth parameters were not significantly impaired up to 20% BSF inclusion, the reduction in Fulton’s condition factor and the intestinal alterations observed in the BSF20 group suggest that this inclusion level may approach a physiological tolerance threshold for this species. The non-significant variation in FCR and SGR values suggests that 15% replacement of dietary conventional protein sources with BSF meal is conceivable in this species. In contrast, low inclusion of BSF meal exerted beneficial effects on intestinal health, thus indicating the role of this ingredient in ameliorating gut defences in this species. The impact of higher percentages of dietary BSF on gut and spleen histology deserves further investigation to assess the ability of flathead grey mullet to digest chitin, and to deepen knowledge on the immune response of this fish species to this novel ingredient.

## Figures and Tables

**Table 1:** Ingredients (g 100 g^−1^) and proximate composition (% as fed on wet weight) of the experimental diets used for the feeding trial.

	BSF0	BSF10	BSF15	BSF20
Ingredients (g 100 g^−1^)				
*Hermetia illucens* meal ^1^	0	4.8	7.2	9.6
Fish meal ^2^	3	1.8	1.2	0.6
Poultry meal ^3^	10	8.8	8.2	7.6
Feather meal (hydrolysate) ^4^	5	3.8	3.2	2.6
Soybean meal ^5^	30	28.8	28.2	27.6
Wheat meal	40	40	40	40
Soybean oil	3	3	3	3
Cod liver oil	3.5	3.5	3.5	3.5
L-lysine	0.85	0.85	0.85	0.85
DL-methionine	0.3	0.3	0.3	0.3
L-tryptophan	0.1	0.1	0.1	0.1
Vitamin e50 ^6^	0.05	0.05	0.05	0.05
Vitamin-mineral premix ^7^	1	1	1	1
Dicalcium phosphate	3	3	3	3
Antioxidant mix ^8^	0.2	0.2	0.2	0.2
Approximate composition (% on dry matter)				
Dry matter	85.85	85.88	85.90	85.91
Crude protein	37.2	36.9	36.6	36.5
Ether extract	9.1	9.5	9.7	9.8
Ash	6.7	6.8	6.6	6.5
Crude fibre	1.5	2.1	2.4	2.6
NFE ^9^	45.5	44.7	44.7	44.6
Gross Energy (MJ/kg feed)	20.24	20.19	20.19	20.19

^1^ Partially defatted *Hermetia illucens* meal (DM, 92.01%; CP, 47.3%; CF, 14.1%; crude fibre, 12.40%; ash, 10.24% as fed); ^2^ fish meal (CP, 59,5%; CF, 11% as fed), Köster Marine Proteins GmbH (Hamburg, Germany); ^3^ poultry meal (CP, 65%; CF, 14.5% as fed), ECB, Saria^®^ (Selm, Germany); ^4^ feather meal (CP, 80.5%; CF, 10% as fed), ECB, Saria^®^; ^5^ high protein soybean meal, non-GMO (CP, 46.5%; CF, 1.5% as fed), BUNGE© (Geneva, Switzerland); ^6^ ROVIMIXE50, DSM Nutritional Products, Grenzach, Germany; ^7^ vitamins and mineral premix (mg kg^−1^ diet, Naturalleva, Cologna Veneta, Italy): D 0.05 mg, A 2.37 mg, E 323.9 mg, inositol 158.0 mg, niacin 182.7 mg, pantothenic acid 67.6 mg, B2 27.4 mg, B1 27.7 mg, B6 24.27 mg, folic acid 6.52 mg, K 5.37 mg, biotin 0.96 mg, B12 0.05 mg, choline 1314.58 mg, C 250.25 mg, calcium 0.72 mg, cobalt 0.30 mg, copper 48.62 mg, iron 494.7 mg, magnesium 21.2 mg, manganese 25.89 mg, molybdate 0.97 mg, nickel 0.80 mg, phosphorus 0.51 mg, potassium 0.83 mg, sodium 0.14 mg, selenium 0.83 mg, sulfur 0.35 mg, zinc 52.7 mg; ^8^ propyl gallate (Naturalleva, Cologna Veneta, Italy): 9.9%; B.H.A., 5.0%; ethoxyquin, 9.9%; citric acid, 11.0%; carrier(=SiO_2_) ad100%; ^9^ nitrogen free extract calculated as 100—(crude protein + ether extract + crude fibre + ash). DM: dry matter; CP: crude protein; CF: crude fat.

**Table 2:** Growth performances, zootechnical, and somatic indexes and survival of *M. cephalus* fed the experimental diets. Different letters in the same row indicate statistically significant differences (*p* < 0.05).

	BSF0	BSF10	BSF15	BSF20	*p* Value
Initial BW (g)	40.00 ± 12.18	40.70 ± 12.72	39.90 ± 12.73	40.30 ± 11.95	
Final BW (g)	95.34 ± 4.53	92.16 ± 1.90	87.17 ± 7.66	82.77 ± 4.27	0.064
FCR	3.28 ± 0.24	3.84 ± 0.50	3.81 ± 0.76	4.34 ± 0.32	0.157
SGR (% day^−1^)	0.62 ± 0.06	0.53 ± 0.07	0.53 ± 0.09	0.49 ± 0.02	0.170
HSI (%)	1.16 ± 0.06	1.06 ± 0.05	1.15 ± 0.04	1.09 ± 0.04	0.087
VSI (%)	7.26 ± 0.36	6.83 ± 0.26	7.35 ± 0.30	7.10 ± 0.13	0.178
K	0.96 ± 0.001 **^a^**	0.95 ± 0.004 **^a^**	0.94 ± 0.019 **^ab^**	0.92 ± 0.009 ^b^	0.019
Survival (%)	99.89 ± 0.19	99.89 ± 0.19	99.56 ± 0.19	99.78 ± 0.38	0.323

Initial and final body weight (BW), feed conversion ratio (FCR), specific growth rate (SGR), hepato-somatic index (HSI), viscero-somatic index (VSI), Fulton condition factor (K) and survival.

**Table 3:** Morphometric evaluations and goblet cell quantification in anterior and posterior intestine of fish fed BSF0, BSF10, BSF15 and BSF20 diets. Different letters indicate statistically significant differences (*p* < 0.05).

	**BSF0**	**BSF10**	**BSF15**	**BSF20**	***p*** **Value**
Anterior intestine					
Villi length (µm)	650.0 ± 179.1	776.6 ± 179.0	820.5 ± 174.6	780.3 ± 151.5	0.097
Villi thickness (µm)	93.0 ± 13.4 ^b^	109.3 ± 10.6 ^a^	110.2 ± 15.2 ^a^	99.7 ± 16.1 ^ab^	0.011
Mucous cells (n)	77.4 ± 21.0 ^b^	97.0 ± 18.4 ^ab^	114.3 ± 26.3 ^a^	88.1 ± 20.7 ^ab^	0.001
Posterior intestine					
Villi length (µm)	498.1 ± 109.6	460.0 ± 88.6	416.7 ± 99.8	409.6 ± 81.2	0.105
Villi thickness (µm)	107.6 ± 22.7	109.2 ± 31.9	98.0 ± 18.5	111.9 ± 24.4	0.559
Mucous cells (n)	133.1 ± 76.1	184.5 ± 81.9	196.3 ± 64.3	188.1 ± 63.9	0.142

**Table 4:** Enteritis score in anterior and posterior intestine of fish fed BSF0, BSF10, BSF15 and BSF20 diets. Different letters indicate statistically significant differences (*p* < 0.05).

	BSF0	BSF10	BSF15	BSF20	*p* Value
Anterior intestine	10.3 ± 2.7 ^b^	10.5 ± 1.9 ^b^	12.1 ± 2.9 ^b^	14.1 ± 2.1 ^a^	0.002
Posterior intestine	14.3 ± 5.5	15.7 ± 4.7	16.9 ± 5.6	15.8 ± 5.8	0.725

**Table 5:** Count of hepatocytes and decentralized nuclei (%) per area from the different experimental groups.

	BSF0	BSF10	BSF15	BSF20	*p* Value
Hepatocyte number/area (76,000 μm^2^)	548 ± 54	542 ± 67	531 ± 39	543 ± 42	0.880
Decentralized nuclei (%)	0.48 ± 0.05	0.84 ± 0.06	0.84 ± 0.04	0.83 ± 0.04	0.732

**Table 6:** Melanomacrophage centre (MMC) count in spleen of fish fed BSF0, BSF10, BSF15 and BSF20 diets. Different letters indicate statistically significant differences (*p* < 0.05).

MMCs (n/area)	BSF0	BSF10	BSF15	BSF20	*p* Value
All sizes	4.8 ± 1.8	5.5 ± 1.2	6.5 ± 4.9	8.0 ± 1.7	0.260
Small	3.7 ± 2.4 ^ab^	1.5 ± 1.2 ^b^	2.2 ± 2.1 ^b^	6.5 ± 2.2 ^a^	0.001
Medium	1.2 ± 2.0	2.5 ± 1.2	3.0 ± 2.9	1.3 ± 0.8	0.302
Large	0.0 ± 0.0 ^b^	1.5 ± 1.0 ^a^	1.3 ± 0.8 ^a^	0.2 ± 0.4 ^b^	0.001

**Table 7:** IL-1β+ and TNF-α+ cell counts in anterior and posterior intestine of fish fed BSF0, BSF10, BSF15 and BSF20 diets. Results are reported as mean ± standard deviation (SD) of the counts. Letters indicate statistically significant differences (*p* < 0.05). No TNF-α+ cells were detected in the posterior intestine of the samples analyzed.

		BSF0	BSF10	BSF15	BSF20	*p* Value
Anterior intestine	IL-1β+ cells	32.7 ± 20.3	44.1 ± 36.1	19.8 ± 7.4	23.2 ± 18.4	0.1134
	TNF-α+ cells	8.1 ± 9.7 ^b^	41.5 ± 28.6 ^a^	16.2 ± 19.3 ^b^	4.4 ± 10.4 ^b^	0.0015
Posterior intestine	IL-1β+ cells	6.8 ± 5.6	0.6 ± 1.3	7.8 ± 11.4	9.5 ± 12.7	0.5428
	TNF-α+ cells	-	-	-	-	-

## Data Availability

The original contributions presented in this study are included in the article/Supplementary Materials. Further inquiries can be directed to the corresponding author.

## Supplementary Materials

The following supporting information can be downloaded at: https://www.mdpi.com/article/10.3390/ani16071012/s1, Table S1. Amino acid (AA) concentration (g 100 g^-1^ of protein) of experimental diets; Table S2. Score assignment criteria for the evaluation of intestine condition; Figure S1. Negative controls for anti-IL-1β (a) and anti-TNF-α antibodies (b). Sections of anterior intestine. Scale bar= 100 µm.

## References

[1] FAO (2024). The State of World Fisheries and Aquaculture 2024—Blue Transformation in Action.

[2] Carballeira Braña C.B., Cerbule K., Senff P., Stolz I.K. (2021). Towards environmental sustainability in marine finfish aquaculture. Front. Mar. Sci..

[3] Jiang Q., Bhattarai N., Pahlow M., Xu Z. (2022). Environmental sustainability and footprints of global aquaculture. Resour. Conserv. Recycl..

[4] Macusi E.D., Cayacay M.A., Borazon E.Q., Sales A.C., Habib A., Fadli N., Santos M.D. (2023). Protein fishmeal replacement in aquaculture: A systematic review and implications on growth and adoption viability. Sustainability.

[5] Serra V., Pastorelli G., Tedesco D.E.A., Turin L., Guerrini A. (2024). Alternative protein sources in aquafeed: Current scenario and future perspectives. Vet. Anim. Sci..

[6] Tacon A., Metian M. (2008). Global overview on the use of fish meal and fish oil in industrially compounded aquafeeds: Trends and future prospects. Aquaculture.

[7] Zhao K., Zhang M., Wang K., Zhu K., Xu C., Xie J., Xu J. (2021). Aquaculture impacts on China’s marine wild fisheries over the past 30 Years. Front. Mar. Sci..

[8] Massa F., Onofri L., Fezzardi D., Nunes P.A.L.D., Svensson L.E., Markandya A. (2017). Aquaculture in the Mediterranean and the Black sea: A blue growth perspective. Handbook on the Economics and Management of Sustainable Oceans.

[9] (2022). FishStatJ—FAO Fishery and Aquaculture Global Statistics. https://www.fao.org/fishery/en/statistics.

[10] Cai J., Chan H.L., Yan X., Leung P. (2023). A global assessment of species diversification in aquaculture. Aquaculture.

[11] Pauly D., Tyedmers P., Froese R., Liu L.Y. (1998). Fishing Down Marine Food Webs. Science.

[12] Tacon A.G., Hasan M.R., Metian M. (2011). Demand and Supply of Feed Ingredients for Farmed Fish and Crustaceans: Trends and Prospects.

[13] Crosetti D., Blaber S.J.M. (2015). Biology, Ecology and Culture of Grey Mullets (Mugilidae).

[14] Whitfield A.K., Panfili J., Durand J.-D. (2012). A global review of the cosmopolitan flathead mullet *Mugil cephalus* Linnaeus 1758 (Teleostei: Mugilidae), with emphasis on the biology, genetics, ecology and fisheries aspects of this apparent species complex. Rev. Fish Biol..

[15] Oren O.H. (1981). Aquaculture of Grey Mullet.

[16] Biswas G., De D., Thirunavukkarasu A.R., Natarajan M., Sundaray J.K., Kailasam M., Kumar P., Ghoshal T.K., Ponniah A.G., Sarkar A. (2012). Effects of stocking density, feeding, fertilization and combined fertilization-feeding on the performances of striped grey mullet (*Mugil cephalus* L.) fingerlings in brackishwater pond rearing systems. Aquaculture.

[17] Essa M.A. (2007). Effect of stocking densities of grey mullet (*Mugil cephalus*) reared on natural food in monoculture earthen ponds on growth performance and total production with economical evaluation. Egypt. J. Aquat. Biol. Fish..

[18] Mondal A., Chakravortty D., Mandal S., Bhattacharyya S.B., Mitra A. (2015). Feeding ecology and prey preference of grey mullet, *Mugil cephalus* (Linnaeus, 1758) in extensive brackish water farming system. J. Marine. Sci. Res. Dev..

[19] FAO (2025). Mugil cephalus. Cultured Aquatic Species Information Programme. Fisheries and Aquaculture. https://www.fao.org/fishery/en/culturedspecies/mugil_cephalus/en.

[20] Bertini A., Natale S., Gisbert E., Andrée K.B., Concu D., Dondi F., De Cesare A., Indio V., Gatta P.P., Bonaldo A. (2023). Exploring the application of *Corynebacterium glutamicum* single cell protein in the diet of flathead grey mullet (*Mugil cephalus*): Effects on growth performance, digestive enzymes activity and gut microbiota. Front. Mar. Sci..

[21] Busti S., Bonaldo A., Dondi F., Cavallini D., Yúfera M., Gilannejad N., Javier Moyano F., Gatta P.P., Parma L. (2020). Effects of different feeding frequencies on growth, feed utilisation, digestive enzyme activities and plasma biochemistry of gilthead sea bream (*Sparus aurata*) fed with different fishmeal and fish oil dietary levels. Aquaculture.

[22] Pelusio N.F., Bonaldo A., Gisbert E., Andree K.B., Esteban M.A., Dondi F., Sabetti M.C., Gatta P.P., Parma L. (2022). Different fish meal and fish oil dietary levels in European Sea bass: Welfare implications after acute confinement stress. Front. Mar. Sci..

[23] De Carvalho C.V.A., Bianchini A., Tesser M.B., Sampaio L.A. (2010). The effect of protein levels on growth, postprandial excretion and tryptic activity of juvenile mullet *Mugil platanus* (Günther). Aquac. Res..

[24] Debasis D., Ghoshal T.K., Kundu J., Ali S.A. (2011). Optimal dietary lipid requirement for grey mullet (*Mugil cephalus*). IJAN.

[25] Debasis D.M., Ghoshal T.K., Kundu J. (2012). Effect of feeding different levels of protein on growth performance, feed utilization and digestive enzyme of grey mullet (*Mugil cephalus* L). Anim. Nutr. Technol..

[26] Talukdar A., Deo A.D., Sahu N.P., Sardar P., Aklakur M., Prakash S., Shamna N., Kumar S. (2020). Effects of dietary protein on growth performance, nutrient utilization, digestive enzymes and physiological status of grey mullet, *Mugil cephalus* L. fingerlings reared in inland saline water. Aquac. Nutr..

[27] Ramos-Júdez S., Duncan N. (2022). Feeding habits and the influence of pellet diameter on feeding responses of flathead grey mullet (*Mugil cephalus*) in captivity. Anim. Feed Sci. Technol..

[28] Abo-Taleb H.A., El-feky M.M.M., Azab A.M., Mabrouk M.M., Elokaby M.A., Ashour M., Mansour A.T., Abdelzaher O.F., Abualnaja K.M., Sallam A.E. (2021). Growth performance, feed utilization, gut integrity, and economic revenue of grey mullet, *Mugil cephalus*, fed an increasing level of dried zooplankton biomass meal as fishmeal substitutions. Fishes.

[29] Gisbert E., Mozanzadeh M.T., Kotzamanis Y., Estévez A. (2016). Weaning wild flathead grey mullet (*Mugil cephalus*) fry with diets with different levels of fish meal substitution. Aquaculture.

[30] Koven W., Gisbert E., Meiri-Ashkenazi I., Nixon O., Israeli D., Tandler A., Nolasco Soria H., Solovyev M., Rosenfeld H. (2020). The effect of weaning diet type on grey mullet (*Mugil cephalus*) juvenile performance during the trophic shift from carnivory to omnivory. Aquaculture.

[31] Wassef E.A., El Masry M.H., Mikhail F.R. (2001). Growth enhancement and muscle structure of striped mullet, *Mugil cephalus* L., fingerlings by feeding algal meal-based diets. Aquac. Res..

[32] El-Dahhar A.A., Salama M.E., Moustafa Y.T., Elmorshedy E.M. (2014). Effect of using equal mixture of seaweeds and marine algae in striped mullet (*Mugil cephalus*) larval diets on growth performance and feed utilization. J. Arab. Aquacult. Soc..

[33] Henry M., Gasco L., Piccolo G., Fountoulaki E. (2015). Review on the use of insects in the diet of farmed fish: Past and future. Anim. Feed Sci. Technol..

[34] Maulu S., Langi S., Hasimuna O.J., Missinhoun D., Munganga B.P., Hampuwo B.M., Gabriel N.N., Elsabagh M., Van Doan H., Abdul Kari Z. (2022). Recent advances in the utilization of insects as an ingredient in aquafeeds: A review. Anim. Nutr..

[35] Caligiani A., Marseglia A., Leni G., Baldassarre S., Maistrello L., Dossena A., Sforza S. (2018). Composition of black soldier fly prepupae and systematic approaches for extraction and fractionation of proteins, lipids and chitin. Food Res. Int..

[36] Makkar H.P.S., Tran G., Heuzé V., Ankers P. (2014). State-of-the-art on use of insects as animal feed. Anim. Feed Sci. Technol..

[37] Smetana S., Schmitt E., Mathys A. (2019). Sustainable use of *Hermetia illucens* insect biomass for feed and food: Attributional and consequential life cycle assessment. Resour. Conserv. Recycl..

[38] Nairuti R.N., Musyoka S.N., Yegon M.J., Opiyo M.A. (2022). Utilization of black soldier fly (*Hermetia illucens*, Linnaeus) larvae as a protein source for fish feed—A review. Aquac. Stud..

[39] Gasco L., Finke M., van Huis A. (2018). Can diets containing insects promote animal health? *J*. Insects Food Feed.

[40] Nogales-Merida S., Gobbi P., Jozefiak D., Mazurkiewicz J., Dudek K., Rawski M., Kieronczyk B., Jozefiak A. (2019). Insect meals in fish nutrition. Rev. Aquac..

[41] Randazzo B., Di Marco P., Zarantoniello M., Daniso E., Cerri R., Finoia M.G., Capoccioni F., Tibaldi E., Olivotto I., Cardinaletti G. (2023). Effects of supplementing a plant protein-rich diet with insect, crayfish or microalgae meals on gilthead sea bream (*Sparus aurata*) and European seabass (*Dicentrarchus labrax*) growth, physiological status and gut health. Aquaculture.

[42] Benhabiles M.S., Salah R., Lounici H., Drouiche N., Goosen M.F.A., Mameri N. (2012). Antibacterial activity of chitin, chitosan and its oligomers prepared from shrimp shell waste. Food Hydrocoll..

[43] Qin C., Zhang Y., Liu W., Xu L., Yang Y., Zhou Z. (2014). Effects of chito-oligosaccharides supplementation on growth performance, intestinal cytokine expression, autochthonous gut bacteria and disease resistance in hybrid tilapia *Oreochromis niloticus* ♀ × *Oreochromis aureus* ♂. Fish Shell. Immunol..

[44] Nawaz A., Javaid A.B., Irshad S., Hoseinifar S.H., Xionga H. (2018). The functionality of prebiotics as immunostimulant: Evidences from trials on terrestrial and aquatic animals. Fish Shellfish Immunol..

[45] Vargas A., Randazzo B., Riolo P., Truzzi C., Gioacchini G., Giorgini E., Loreto N., Ruschioni S., Zarantoniello M., Antonucci M. (2018). Rearing zebrafish on black soldier fly (*Hermetia illucens*): Biometric, histological, spectroscopic, biochemical, and molecular implications. Zebrafish.

[46] Kumar V., Fawole F.J., Romano N., Hossain M.S., Labh S.N., Overturf K., Small B.C. (2021). Insect (black soldier fly, *Hermetia illucens*) meal supplementation prevents the soybean meal-induced intestinal enteritis in rainbow trout and health benefits of using insect oil. Fish Shellfish Immunol..

[47] Singha K.P., Abanikannda M.F., Ma J., Romano N., Koutsos E., Adams D., Kumar V. (2025). Complementing the high soybean meal diet with black soldier fly larvae meal as a functional feed ingredient to improve the performance, nutrient profile, and gut health of rainbow trout, *Oncorhynchus mykiss*. Aquaculture.

[48] Bruni L., Pastorelli R., Viti C., Gasco L., Parisi G. (2018). Characterisation of the intestinal microbial communities of rainbow trout (*Oncorhynchus mykiss*) fed with *Hermetia illucens* (black soldier fly) partially defatted larva meal as partial dietary protein source. Aquaculture.

[49] Belghit I., Liland N., Gjesdal P., Biancarosa I., Menchetti E., Li Y., Waagbø R., Krogdahl Å., Lock E.-J. (2019). Black soldier fly larvae meal can replace fish meal in diets of sea-water phase Atlantic salmon (*Salmo salar*). Aquaculture.

[50] Kroeckel S., Harjes A.-G.E., Roth I., Katz H., Wuertz S., Susenbeth A., Schulz C. (2012). When a turbot catches a fly: Evaluation of a pre-pupae meal of the black soldier fly (*Hermetia illucens*) as fish meal substitute—Growth performance and chitin degradation in juvenile turbot (*Psetta maxima*). Aquaculture.

[51] Fischer H., Romano N., Renukdas N., Kumar V., Kumar Sinha A. (2022). Comparing black soldier fly (*Hermetia illucens*) larvae versus prepupae in the diets of largemouth bass, *Micropterus salmoides*: Effects on their growth, biochemical composition, histopathology, and gene expression. Aquaculture.

[52] Li S., Ji H., Zhang B., Zhou J., Yu H. (2017). Defatted black soldier fly (*Hermetia illucens*) larvae meal in diets for juvenile Jian carp (*Cyprinus carpio* var. Jian): Growth performance, antioxidant enzyme activities, digestive enzyme activities, intestine and hepatopancreas histological structure. Aquaculture.

[53] Paredes J.F., Riche M., Bradshaw D., Mejri S., Chin L.S., Perez J., Popa R., Romano N., Wills P.S. (2025). Evaluation of black soldier fly (*Hermetia illucens* L.) larvae meal in diets of Red drum (*Sciaenops ocellatus*) juvenile production performance and feed palatability. Front. Aquac..

[54] Tippayadara N., Dawood M.A.O., Krutmuang P., Hoseinifar S.H., Doan H.V., Paolucci M. (2021). Replacement of fish meal by black soldier fly (*Hermetia illucens*) larvae meal: Effects on growth, haematology, and skin mucus immunity of Nile tilapia, *Oreochromis niloticus*. Animals.

[55] Zhou J.S., Liu S.S., Ji H., Yu H.B. (2017). Effect of replacing dietary fish meal with black soldier fly larvae meal on growth and fatty acid of Jian carp (*Cyprinus carpio* var. Jian). Aquac. Nutr..

[56] Randazzo B., Zarantoniello M., Cardinaletti G., Cerri R., Giorgini E., Belloni A., Contò M., Tibaldi E., Olivotto I. (2021). *Hermetia illucens* and poultry by-product meals as alternatives to plant protein sources in gilthead seabream (*Sparus aurata*) diet: A multidisciplinary study on fish gut status. Animals.

[57] Mohan K., Rajan D.K., Muralisankar T., Ganesan A.R., Sathishkumar P., Revathi N. (2022). Use of black soldier fly (Hermetia illucens L.) larvae meal in aquafeeds for a sustainable aquaculture industry: A review of past and future needs. Aquaculture.

[58] Gai F., Cusimano G.M., Maricchiolo G., Caccamo L., Caimi C., Macchi M., Meola M., Perdichizzi A., Tartarisco G., Gasco L. (2023). Defatted black soldier fly meal in diet for grow-out gilthead seabream (*Sparus aurata* L. 1758): Effects on growth performance, gill cortisol level, digestive enzyme activities, and intestinal histological structure. Aquac. Res..

[59] Di Rosa A., Caccamo L., Pansera L., Oteri M., Chiofalo B., Maricchiolo G. (2023). Influence of *Hermetia illucens* larvae meal dietary inclusion on growth performance, gut histological traits and stress parameters in *Sparus aurata*. Animals.

[60] AOAC—Association of Official Analytical Chemist (2020). Official Methods of Analysis.

[61] AOAC—Association of official Analytical Chemist (2003). Official Methods of Analysis of the Association of Official’s Analytical Chemists.

[62] Uràn P.A., Gonçalves A.A., Taverne-Thiele J.J., Schrama J.W., Verreth J.A.J., Rombout J.H.W.M. (2008). Soybean meal induces intestinal inflammation in common carp (*Cyprinus carpio* L.). Fish Shellfish. Immunol..

[63] Penn M.H., Bendiksen E.A., Campbell P., Krogdahl A. (2011). High level of dietary pea protein concentrate induces enteropathy in Atlantic salmon (*Salmo salar* L.). Aquaculture.

[64] García-Ortega A., Kissinger K.R., Trushenski J.T. (2016). Evaluation of fish meal and fish oil replacement by soybean protein and algal meal from *Schizochytrium limacinum* in diets for giant grouper *Epinephelus lanceolatus*. Aquaculture.

[65] Mazumder S.K., Das S.K., Bakar Y., Ghaffar M.A. (2016). Effects of temperature and diet on length-weight relationship and condition factor of the juvenile Malabar blood snapper (*Lutjanus malabaricus* Bloch & Schneider, 1801). J. Zhejiang Univ. Sci. B..

[66] Kop A., Korkut A.Y., Gurkan S. (2019). Length-weight relationship and condition factor as an indicator of growth and feeding intensity of Sea bream (*Sparus aurata* L, 1758) given feed with different protein contents. Indian J. Anim. Res..

[67] Hua K. (2021). A meta-analysis of the effects of replacing fish meals with insect meals on growth performance of fish. Aquaculture.

[68] Spranghers T., Ottoboni M., Klootwijk C., Ovyn A., Deboosere S., De Meulenaer B., Michiels J., Eeckhout M., De Clercq P., De Smet S. (2017). Nutritional composition of black soldier fly (*Hermetia illucens*) prepupae reared on different organic waste substrates. J. Sci. Food Agric..

[69] Randazzo B., Zarantoniello M., Gioacchini G., Cardinaletti G., Belloni A., Giorgini E., Faccenda F., Cerri R., Tibaldi E., Olivotto I. (2021). Physiological response of rainbow trout (*Oncorhynchus mykiss*) to graded levels of *Hermetia illucens* or poultry by-product meals as single or combined substitute ingredients to dietary plant proteins. Aquaculture.

[70] Pascon G., Opere Akinyi R., Cardinaletti G., Daniso E., Messina M., Tulli F. (2025). Chitin and its effects when included in aquafeed. Aquac. Int..

[71] Romano N., Datta S.N., Pande G.S.J., Sinha A.K., Yamamoto F., Rawles S.D., Webster C.D. (2025). Preliminary assessment of the nutritive value of dietary exuviae from black soldier fly (*Hermetia illucens*) pupae in Mozambique tilapia. J. World Aquac. Soc..

[72] Tran H.Q., Tram N.T., Prokešová M., Gebauer T., Doan V., Stejskal V., Doan H.V. (2022). Systematic review and meta-analysis of production performance of aquaculture species fed dietary insect meals. Rev. Aquac..

[73] Guerreiro I., Serra C.R., Coutinho F., Couto A., Castro C., Rangel F., Peres H., Pousão-Ferreira P., Matos E., Gasco L. (2021). Digestive enzyme activity and nutrient digestibility in meagre (*Argyrosomus regius*) fed increasing levels of black soldier fly meal (*Hermetia illucens*). Aquacult. Nutr..

[74] Weththasinghe P., Lagos L., Cortés M., Hansen J.Ø., Øverland M. (2021). Dietary inclusion of black soldier fly (*Hermetia illucens*) larvae meal and paste improved gut health but had minor effects on skin mucus proteome and immune response in Atlantic salmon (*Salmo salar*). Front. Immunol..

[75] Shiau S.Y., Yu Y.P. (1999). Dietary supplementation of chitin and chitosan depresses growth in tilapia, *Oreochromis niloticus* x *O-aureus*. Aquaculture.

[76] Alegbeleye W.O., Obasa S.O., Olude O.O., Otubu K., Jimoh W. (2012). Preliminary evaluation of the nutritive value of the variegated grasshopper (*Zonocerus variegatus* L.) for African catfish *Clarias gariepinus* (Burchell. 1822) fingerlings. Aquac. Res..

[77] Rust M.B., Halver J.E., Hardy R.W. (2003). Nutritional Physiology.

[78] Hasan I., Gai F., Cirrincione S., Rimoldi S., Saroglia G., Terova G. (2023). Chitinase and insect meal in aquaculture nutrition: A comprehensive overview of the latest achievements. Fishes.

[79] NRC—National Research Council (2011). Nutrient Requirements of Fish and Shrimp.

[80] Lall S., Dumas A., Davis D.A. (2022). 3—Nutritional requirements of cultured fish: Formulating nutritionally adequate feeds. Woodhead Publishing Series in Food Science, Technology and Nutrition, Feed and Feeding Practices in Aquaculture (Second Edition).

[81] Luzzana U., Valfrè F., Mangiarotti M., Domeneghini C., Radaelli G., Maria Moretti V., Scolari M. (2005). Evaluation of different protein sources in fingerling grey mullet *Mugil cephalus* practical diets. Aquacult. Int..

[82] Vargas-Abúndez A.J., Randazzo B., Foddai M., Sanchini L., Truzzi C., Giorgini E., Gasco L., Olivotto I. (2019). Insect meal based diets for clownfish: Biometric, histological, spectroscopic, biochemical and molecular implications. Aquaculture.

[83] Zarantoniello M., Randazzo B., Secci G., Notarstefano V., Giorgini E., Lock E.J., Parisi G., Olivotto I. (2022). Application of laboratory methods for understanding fish responses to black soldier fly (*Hermetia illucens*) based diets. J. Ins. Food Feed.

[84] Cardinaletti G., Randazzo B., Messina M., Zarantoniello M., Giorgini E., Zimbelli A., Bruni L., Parisi G., Olivotto I., Tulli F. (2019). Effects of graded dietary inclusion level of full-fat *Hermetia illucens* prepupae meal in practical diets for rainbow trout (*Oncorhynchus mykiss*). Animals.

[85] Hender A., Siddik M.A., Howieson J., Fotedar R. (2021). Black soldier fly, Hermetia illucens as an alternative to fishmeal protein and fish oil: Impact on growth, immune response, mucosal barrier status, and flesh quality of juvenile barramundi, *Lates calcarifer* (Bloch, 1790). Biology.

[86] Chaklader M.R., Howieson J., Fotedar R., Siddik M.A. (2021). Supplementation of *Hermetia illucens* larvae in poultry by-product meal-based barramundi, *Lates calcarifer* diets improves adipocyte cell size, skin barrier functions, and immune responses. Front. Nutr..

[87] Zapata A.G. (2024). The fish spleen. Fish Shellfish. Immunol..

[88] Agius C., Roberts R.J. (2003). Melano-macrophage centres and their role in fish pathology. J. Fish Dis..

[89] Terova G., Rimoldi S., Ascione C., Gini E., Ceccotti C., Gasco L. (2019). Rainbow trout (*Oncorhynchus mykiss*) gut microbiota is modulated by insect meal from *Hermetia illucens* prepupae in the diet. Rev. Fish Biol. Fish..

[90] Sayed R.K.A., Zaccone G., Capillo G., Albano M., Mokhtar D.M. (2022). Structural and functional aspects of the spleen in molly fish *Poecilia sphenops* (Valenciennes, 1846): Synergistic interactions of stem cells, neurons, and immune cells. Biology.

[91] Jiang B., Sun Y., Li W., Liu C., Wen C., Li A., Huang Y., Su Y. (2022). Effects of dietary black soldier fly (*Hermetia illucens* Linnaeus) on the disease resistance of juvenile grouper (*Epinephelus coioides*). Fish Shellfish. Immunol..

[92] Krogdahl Å., Kortner T.M., Løkka G., Kumar V. (2025). Chapter 14—Nutrition and the immune system in fish and shellfish. Feed and Feeding for Fish and Shellfish.

[93] Hodgkinson J.W., Grayfer L., Belosevic M. (2015). Biology of Bony Fish Macrophages. Biology.

[94] Wiegertjes G.F., Wentzel A.S., Spaink H.P., Elks P.M., Fink I.R. (2016). Polarization of immune responses in fish: The ’macrophages first’ point of view. Mol. Immunol..

[95] Linh N.V., Wannavijit S., Tayyamath K., Dinh-Hung N., Nititanarapee T., Sumon M.A.A., Srinual O., Permpoonpattana P., Doan H.V., Brown C.L. (2024). Black soldier fly (*Hermetia illucens*) larvae meal: A sustainable alternative to fish meal proven to promote growth and immunity in Koi Carp (*Cyprinus carpio* var. koi). Fishes.

[96] Agulló E., Rodríguez M.S., Ramos V., Albertengo L. (2003). Present and future role of chitin and chitosan in food. Macromol. Biosci..

[97] Kim S.-K., Rajapakse N. (2006). Enzymatic production and biological activities of chitosan oligosaccharides (COS): A review. Carbohydr. Polym..

[98] Kono M., Matsui T., Shimizu C. (1987). Effect of chitin, chitosan, and cellulose as diet supplements on the growth of cultured fish. Nippon Suisan Gakkaishi.

[99] Henry M.A., Gasco L., Chatzifotis S., Piccolo G. (2018). Does dietary insect meal affect the fish immune system? The case of mealworm, *Tenebrio molitor* on European sea bass, *Dicentrarchus labrax*. Dev. Comp. Immunol..

[100] Gasco L., Acuti G., Bani P., Dalle Zotte A., Danieli P.P., De Angelis A., Fortina R., Marino R., Parisi G., Piccolo G. (2020). Insect and fish by-products as sustainable alternatives to conventional animal proteins in animal nutrition. Ital. J. Anim. Sci..

[101] Basili M., Randazzo B., Caccamo L., Guicciardi S., Meola M., Perdichizzi A., Quero G.M., Maricchiolo G. (2025). Effect of graded inclusion of black soldier fly (*Hermetia illucens*, Linnaeus, 1758) pre-pupae meal in diets for gilthead seabream (*Sparus aurata*, Linnaeus, 1758) on gut microbiome and liver morphology. Fish Physiol. Biochem..

[102] Abdel-Latif H.M., Abdel-Tawwab M., Khalil R.H., Metwally A.A., Shakweer M.S., Ghetas H.A., Khallaf M.A. (2021). Black soldier fly (*Hermetia illucens*) larvae meal in diets of European seabass: Effects on antioxidative capacity, non-specific immunity, transcriptomic responses, and resistance to the challenge with *Vibrio alginolyticus*. Fish Shellfish Immunol..

[103] Varaeva Y.R., Kirichenko T.V., Shaposhnikova N.N., Nikityuk D.B., Starodubova A.V. (2022). The role of diet in regulation of macrophages functioning. Biomedicines.

[104] Ali M.M., Elashry M.A., Mohammady E.Y., Soaudy M.R., El-Garhy H.S., El-Erian M.A., Mustafa A., Abouelsoud M., Ragaza J.A., El-Haroun E.R. (2023). Dietary alpha-monolaurin for Nile tilapia (*Oreochromis niloticus*): Stimulatory effects on growth, immunohematological indices, and immune-related gene expressions. Aquac. Res..

[105] Ullah S., Feng F., Zhao M., Zhang J., Shao Q. (2025). Effect of dietary supplementation of lauric acid on growth performance, digestive enzymes, serum immune and antioxidant parameters, and intestinal morphology in black sea bream. Fish Physiol. Biochem..

