# Supplementary material for: Defatted Black Soldier Fly Meal as a Dietary Protein Source for Grey Mullet (Mugil cephalus): Effects on Growth Performance, Gut Morphology, Spleen and Liver Health
Source: Animals (Basel). 2026 Mar 25;16(7):1012. doi: 10.3390/ani16071012 (PMC13072009; doi:10.3390/ani16071012)
Supplement: Supplementary file 1 [file animals-16-01012-s001.zip › Table S2.pdf]

PARAMETER	SCORE	DESCRIPTION
**Oedema**	0	Undetected
	1	Basal level - Up to 2 villi per section
	2	Medium gravity - Up to 4 villi per section
	3	High gravity - More than 4 villi per section
**Epithelium detachment**	0	Undetected
	1	Basal level - Spread epithelial cells in the lumen
	2	Medium gravity - Visible epithelial detachment from the villi's tip
	3	High gravity - Extensive mucosal architecture impairment
**Enterocytes nuclei delocalization**	0	Undetected
	1	Basal level - Occasional dealignment
	2	Medium gravity - Observed in approximately 50% villi
	3	High gravity - Extensively observed
**Enterocytes supranuclear vacuolization**	0	Undetected - Small and regular scattered vacuoles
	1	Basal level - Big vacuoles, scattered along the mucosal layer
	2	Medium gravity - Big vacuoles, diffused along the mucosal layer
	3	High gravity - Big vacuoles, diffused and enterocyte congestion
**Submucosa thickening**	0	~ 5-10 μm
	1	~ 15-20 μm
	2	~ 25-30 μm
	3	> 30 μm
**Lamina propria thickening**	0	~ 2-5 μm
	1	~ 10 - 20μm
	2	~ 25-30 μm
	3	> 30 μm
**Inflammatory infiltrate**	0	Scarce lymphocyte infiltration
	1	Moderate infiltration
	2	Diffused infiltration
	3	Highly infiltrated

Table 2S. Score assignment criteria for the evaluation of intestine condition.
