# Supplementary material for: Defatted Black Soldier Fly Meal as a Dietary Protein Source for Grey Mullet (Mugil cephalus): Effects on Growth Performance, Gut Morphology, Spleen and Liver Health
Source: Animals (Basel). 2026 Mar 25;16(7):1012. doi: 10.3390/ani16071012 (PMC13072009; doi:10.3390/ani16071012)
Supplement: Supplementary file 1 [file animals-16-01012-s001.zip › Table S1.pdf]

Essential AA				
Arginine	8.7	8.5	8.4	8.2
Histidine	2.9	3.0	3.0	3.0
Isoleucine	5.5	5.5	5.4	5.4
Leucine	9.3	9.1	9.0	8.9
Lysine	6.9	6.9	6.9	6.8
Methionine	2.0	2.0	2.0	2.0
Cysteine	2.3	2.1	2.0	1.9
Phenylalanine	5.9	5.9	5.8	5.8
Tyrosine	4.0	4.2	4.3	4.4
Threonine	4.7	4.6	4.5	4.5
Tryptophan	1.5	1.5	1.4	1.4
Valine	6.3	6.3	6.3	6.3

Table 1S. Amino acid (AA) concentration (g 100 g^{-1} of protein) of experimental diets
