# Supplementary figures and images for: Defatted Black Soldier Fly Meal as a Dietary Protein Source for Grey Mullet (Mugil cephalus): Effects on Growth Performance, Gut Morphology, Spleen and Liver Health
Source: Animals (Basel). 2026 Mar 25;16(7):1012. doi: 10.3390/ani16071012 (PMC13072009; doi:10.3390/ani16071012)

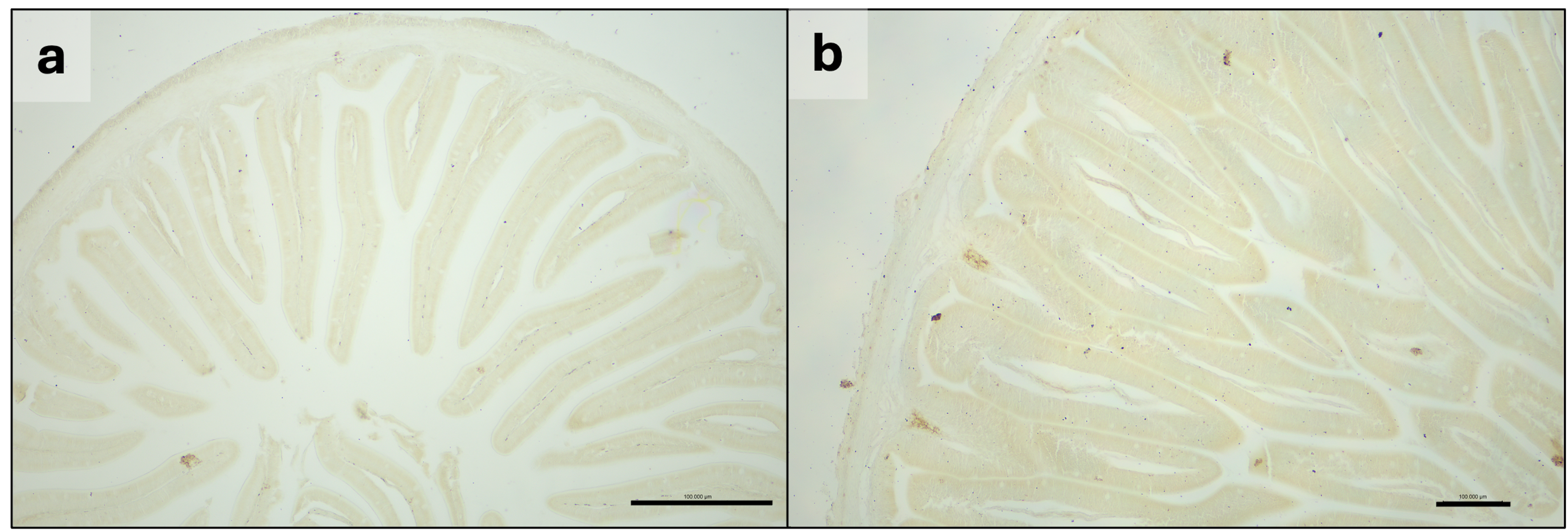

Supplement: Supplementary file 1 [file animals-16-01012-s001.zip › Fig. S1.tif]
